# The Silent Epidemic—Chronic Pain and Palliative Care Needs in Children and Adolescents

**DOI:** 10.3390/children11030283

**Published:** 2024-02-25

**Authors:** Boris Zernikow

**Affiliations:** 1 German Paediatric Pain Centre, Children’s and Adolescents’ Hospital, 45711 Datteln, Germany; b.zernikow@kinderklinik-datteln.de; Tel.: +49-2363-975180; 2 Department of Children’s Pain Therapy and Paediatric Palliative Care, Faculty of Health, School of Medicine, Witten/Herdecke University, 58448 Witten, Germany

As the Section Editor-in-Chief, it is my pleasure to introduce the new section of *Children* dedicated to pediatric pain and palliative care.

Worldwide, the treatment of acute and chronic pain is an unmet need in children and adolescents [1,2]. Health disparities, especially racial and ethnic disparities, have been identified in all fields of pediatric pain management, exacerbating the challenges in addressing this issue [3]. This new section is intended for pain clinicians, educators, and researchers interested in pain from various medical and psychological specialties and related healthcare areas. 

In the field of acute pain, the focus lies on neglected areas in research and pain treatment, such as vaccination pain [4], acute pain in other healthcare settings, and acute pain escalation in the trajectory of severe illnesses [5,6].

Another crucial field covered is chronic pain in children—encompassing symptoms like headache, abdominal pain, and musculoskeletal pain—which may arise primarily or secondarily during episodes of organ destruction or malfunction. Around 30% of all schoolchildren suffer from primary chronic pain, with 8% facing chronic pain conditions associated with serious emotional or functional impairment (contribution 1) [7,8]. Children with severe pain disorders may experience reduced developmental potential [9], and chronic pain in adolescents often leads to chronic pain in adulthood (contribution 2). For many children, multimodal treatment is effective [10,11] and can reduce overall healthcare costs (contribution 3). However, several research questions remain unanswered. How do we tailor treatment to the individual needs of children? Are there therapy components that can be delivered remotely? How can chronic pain be effectively prevented in schoolchildren?

Pediatric Palliative Care focuses on children, adolescents, and young adults with life-threatening or life-limiting conditions and their families throughout the entire disease trajectory from diagnosis to death. Approximately 21 million children worldwide are deemed in need of a pediatric palliative care approach [12]. Over the past 20 years, there has been a rise in the number of pediatric patients with severe chronic diseases and palliative care needs [13]. Patients’ ages range between zero and 30 years. Often, they suffer from rare diseases with a focus on neurological, congenital, metabolic, oncological, or as of yet undiagnosed/unknown conditions [14,15]. Many patients are non-verbal (contribution 4) [14,15] and may present with a variety of heterogeneous and interrelated symptoms (contribution 5), including pain, irritability of unknown origin (contribution 6), sleep problems (contribution 7), or dyspnea (contribution 8).

Communication with patients and parents [16], shared decision making [17], advanced care planning [18], and multimodal and interprofessional treatment [19] are major topics of interest. This new section welcomes articles on the four dimensions of palliative care: biological, psychological, social, and spiritual.

The Pediatric Pain Therapy and Palliative Care Section invites submissions of articles and reviews related to pediatric pain and palliative care.

## References

[1] Pillai Riddell R.R., Bucsea O., Shiff I., Chow C., Gennis H.G., Badovinac S., DiLorenzo-Klas M., Racine N.M., Ahola Kohut S., Lisi D. (2023). Non-pharmacological management of infant and young child procedural pain. Cochrane Database Syst. Rev..

[2] Kleinhans A. (2023). Acute Pain Management Protocols in Pediatric Intensive Care Units. Crit. Care Nurs. Clin. North Am..

[3] Nguyen L.H., Dawson J.E., Brooks M., Khan J.S., Telusca N. (2023). Disparities in Pain Management. Anesthesiol. Clin..

[4] Taddio A., Rogers J.M. (2015). Why are children still crying? Going beyond “evidence” in guideline development to improve pain care for children: The HELPinKIDS experience. Pain.

[5] Shearer H.M., Verville L., Côté P., Hogg-Johnson S., Fehlings D.L. (2023). Clinical course of pain intensity in individuals with cerebral palsy: A prognostic systematic review. Dev. Med. Child Neurol..

[6] Verschueren S., van Aalst J., Bangels A.-M., Toelen J., Allegaert K., Buffel C., Vander Stichele G. (2019). Development of CliniPup, a Serious Game Aimed at Reducing Perioperative Anxiety and Pain in Children: Mixed Methods Study. JMIR Serious Games.

[7] Sommer A., Grothus S., Grochowska K., Claus B.B., Stahlschmidt L., Wager J. (2022). Assessing fatigue in children and adolescents: Psychometric validation of the German version of the PROMIS® Pediatric Short Form v2.0—Fatigue 10a in school children and pediatric chronic pain patients. Qual. Life Res..

[8] Könning A., Rosenthal N., Brown D., Stahlschmidt L., Wager J. (2021). Severity of Chronic Pain in German Adolescent School Students: A Cross-sectional Study. Clin. J. Pain.

[9] Ragnarsson S., Myleus A., Hurtig A.-K., Sjöberg G., Rosvall P.-Å., Petersen S. (2020). Recurrent Pain and Academic Achievement in School-Aged Children: A Systematic Review. J. Sch. Nurs..

[10] Claus B.B., Stahlschmidt L., Dunford E., Major J., Harbeck-Weber C., Bhandari R.P., Baerveldt A., Neß V., Grochowska K., Hübner-Möhler B. (2022). Intensive interdisciplinary pain treatment for children and adolescents with chronic noncancer pain: A preregistered systematic review and individual patient data meta-analysis. Pain.

[11] Liossi C., Johnstone L., Lilley S., Caes L., Williams G., Schoth D.E. (2019). Effectiveness of interdisciplinary interventions in paediatric chronic pain management: A systematic review and subset meta-analysis. Br. J. Anaesth..

[12] Connor S.R., Downing J., Marston J. (2017). Estimating the Global Need for Palliative Care for Children: A Cross-sectional Analysis. J. Pain Symptom Manag..

[13] Fraser L.K., Gibson-Smith D., Jarvis S., Norman P., Parslow R.C. (2021). Estimating the current and future prevalence of life-limiting conditions in children in England. Palliat. Med..

[14] Hoell J.I., Warfsmann J., Gagnon G., Trocan L., Balzer S., Oommen P.T., Borkhardt A., Janßen G., Kuhlen M. (2017). Palliative care for children with a yet undiagnosed syndrome. Eur. J. Pediatr..

[15] Hoell J.I., Weber H., Warfsmann J., Trocan L., Gagnon G., Danneberg M., Balzer S., Keller T., Janßen G., Kuhlen M. (2019). Facing the large variety of life-limiting conditions in children. Eur. J. Pediatr..

[16] Feraco A.M., Dussel V., Orellana L., Kang T.I., Geyer J.R., Rosenberg A.R., Feudtner C., Wolfe J. (2017). Tumor Talk and Child Well-Being: Perceptions of “Good” and “Bad” News Among Parents of Children with Advanced Cancer. J. Pain Symptom Manag..

[17] Dreesens D., Veul L., Westermann J., Wijnands N., Kremer L., van der Weijden T., Verhagen E. (2019). The clinical practice guideline palliative care for children and other strategies to enhance shared decision-making in pediatric palliative care; pediatricians’ critical reflections. BMC Pediatr..

[18] Lusney N., van Breemen C., Lim E., Pawliuk C., Hussein Z. (2023). Pediatric Advance Care Planning: A Scoping Review. Children.

[19] Peláez-Cantero M.J., Morales-Asencio J.M., Navarro-Mingorance Á., Madrid-Rodriguez A., Tavera-Tolmo Á., Escobosa-Sánchez O., Martino-Alba R. (2023). End of life in patients attended by pediatric palliative care teams: What factors influence the place of death and compliance with family preferences?. Eur. J. Pediatr..

